# Influence of Enhanced Recovery Pathway on Surgical Site Infection after Colonic Surgery

**DOI:** 10.1155/2017/9015854

**Published:** 2017-10-31

**Authors:** Caroline Gronnier, Fabian Grass, Christiane Petignat, Basile Pache, Dieter Hahnloser, Giorgio Zanetti, Nicolas Demartines, Martin Hübner

**Affiliations:** ^1^Department of Visceral Surgery, Lausanne University Hospital (CHUV), Lausanne, Switzerland; ^2^Department of Hospital Preventive Medicine, Lausanne University Hospital (CHUV), Lausanne, Switzerland

## Abstract

**Background:**

The present study aimed to evaluate a potential effect of ERAS on surgical site infections (SSI).

**Methods:**

Colonic surgical patients operated between May 2011 and September 2015 constituted the cohort for this retrospective analysis. Over 100 items related to demographics, surgical details, compliance, and outcome were retrieved from a prospectively maintained database. SSI were traced by an independent National surveillance program. Risk factors for SSI were identified by univariate and multinomial logistic regression.

**Results:**

Fifty-four out of 397 patients (14%) developed SSI. Independent risk factors for SSI were emergency surgery (OR 1.56; 95% CI 1.09–1.78, *p* = 0.026), previous abdominal surgery (OR 1.7; 95% CI 1.32–1.87, *p* = 0.004), smoking (OR 1.71; 95% CI 1.22–1.89, *p* = 0.014), and oral bowel preparation (OR 1.86; 95% CI 1.34–1.97, *p* = 0.013), while minimally invasive surgery (OR 0.3; 95% CI 0.16–0.56, *p* < 0.001) protected against SSI. Compliance to ERAS items of >70% was not retained as a protective factor for SSI after multivariate analysis (OR 0.94; 95% CI 0.46–1.92, *p* = 0.86).

**Conclusions:**

Smoking, open and emergency surgery, and bowel preparation were risk factors for SSI. ERAS pathway had no independent impact while minimally invasive approach did. This study was registered under ResearchRegistry.com (UIN researchregistry2614).

## 1. Introduction

Enhanced recovery after surgery (ERAS) pathways aim to reduce surgical stress [1]. In colorectal surgery, ERAS has been associated with decreased complication rates, positively influencing length of stay and costs [2, 3]. Thus, ERAS represents a fundamental shift in perioperative care.

Surgical site infections (SSI) occur in up to 22% of patients and depend on patient- and surgery-related factors [4]. In Switzerland, SSI rate of 18.2% after colectomy has been described recently in a large prospective surveillance study [5].

SSI increase hospital readmission rates [6, 7] and health costs [8] and delay adjuvant chemotherapy [9]. Appropriate choice and timing of antibiotic prophylaxis, normothermia, and perioperative normoglycemia have been identified as protective factors against SSI [10, 11]. Several implementation programs for evidence-based practice have demonstrated a decrease of SSI applying these principles in colorectal surgery [12, 13], and these items are part of ERAS guidelines for colorectal surgery [3].

The aim of the present study was to assess incidence and risk factors for SSI in a cohort of colonic surgery patients treated within an ERAS pathway.

## 2. Methods

### 2.1. Patients

All consecutively operated colonic surgical patients between May 2011 and September 2015 at the Lausanne University Hospital (CHUV) were included in the analysis. All patients were treated within a standardized ERAS pathway [14]. Open and laparoscopic colectomies in an elective or emergency (since April 2012) setting were included. The only exclusion criterium was documented anastomotic leakage in order to avoid confounding factor for SSI.

This study was approved by the Institutional Review Board (Commission cantonale d'éthique de la recherche sur l'être humain CER-VD, # 2016–00991). The study was conducted according to the STROBE criteria and registered under ResearchRegistry.com (UIN researchregistry2614).

Demographic and surgical information was prospectively assessed in a dedicated database by the specialized ERAS nurse; accuracy of data entry was cross-checked by independent review during Hebdomadal audits. Data about ERAS-specific perioperative care items were prospectively recorded [3]. Demographic information included age, gender, body mass index (BMI), American Society of Anesthesiologists (ASA) score, and smoking status (daily smoker versus nonsmoker) at the time of the procedure. Further recorded variables were diagnoses including the presence of malignancy, immunosuppressive treatments (i.e., steroids) by the time of the procedure, neoadjuvant chemotherapy, drug-requiring diabetes mellitus, and previous abdominal surgery. Surgical information included type of procedure (sigmoid, left, right, or total colectomy (excluding proctocolectomy) and Hartmann reversal), approach (open versus laparoscopy with conversion assigned to laparoscopy according to the *intention-to-treat* principle), setting (elective versus emergency within 72 hours after unplanned admission), duration, anastomotic technique (hand-sewn, circular, or linear stapler), and confection of a new stoma (both colo- or ileostomy). According to the Institutional guidelines, intravenous cefuroxime 1.5 g and metronidazole 500 mg were systematically administered 60–30 min before incision. As an alternative in case of nontolerance, clindamycin 600 mg and ciprofloxacin 400 mg were used. Compliance to cefuroxime administration and timing of administration were analyzed.

### 2.2. Assessment of Compliance to ERAS Items

*Overall compliance* to 19 pre-, peri-, and postoperative ERAS care items was assessed and stratified with a cutoff of 70% according to the previous publications [15, 16]. These items were preadmission patient education, no oral bowel preparation, preoperative oral carbohydrate drinks, no preoperative long-acting sedative medication, thromboprophylaxis, antibiotic prophylaxis, postoperative nausea and vomiting (PONV) prophylaxis (droperidol 1 mg, ondansetron 4 mg, and bethamethasone 4 mg), intraoperative thoracic epidural analgesia, hypothermia prevention (active warming by air blanket), fluid administration guidance, balanced intravenous fluids (ml/kg/h < 7), no prophylactic nasogastric tube, no abdominal drain, sip feeds at postoperative day (POD) 0 of >300 kcal, sufficient oral fluids (>1 L) at POD 0, systematic laxatives, IV fluid administration at POD 1 of <500 mL, weight gain of <1.5 kg at POD 1, and mobilization at all at POD 0.

### 2.3. Assessment and Classification of Surgical Site Infection

SSI data were prospectively monitored through an in-hospital and postdischarge National surveillance program (systematic phone call at postoperative day (POD) 30) by an independent committee (http://www.swissnoso.ch). Methodological details of this assessment have been published previously [5]. SSI were classified according to the Center for Disease Control (CDC) National Nosocomial Infection Surveillance (NNIS) criteria into superficial incisional, deep incisional, and organ space infections [17]. Contamination class was assessed by the surgeon and classified at the end of the procedure as clean contaminated (grade II), contaminated (grade III), or infectious (grade IV). A second independent assessment (NNIS score 0–3) was performed by the surveillance committee according to NNIS criteria based on ASA score, wound class (independently assessed based on surgery reports and stratified likewise (II–IV)), and duration of surgery.

### 2.4. Outcomes/Study Endpoints

The primary endpoint was the rate of SSI. Uni- and multivariate risk factors for SSI were identified among demographic, surgery-related, and perioperative ERAS care items. Modifiable pre- and perioperative ERAS items, overall compliance to the ERAS pathway, readmission rates, and length of stay were compared between the two groups (SSI versus no SSI).

### 2.5. Statistical Analysis

Descriptive statistics for categorical variables were reported as frequency (%), while continuous variables were reported as mean (standard deviation) or median (interquartile range). Chi-square was used for comparison of categorical variables. All statistical tests were two-sided, and a level of 0.05 was used to indicate statistical significance. Variables with *p* values ≤0.05 including potential confounding factors (ERAS compliance >70%, smoking status) were then entered into a multivariate logistic regression (based on a probit regression model) to provide adjusted estimations of the odds ratio (OR). Data analysis was performed with the Statistical Software for the Social Sciences SPSS Advanced Statistics 22 (IBM Software Group, 200 W. Madison St., Chicago, IL; 60606 USA).

## 3. Results

### 3.1. Patients

Out of 413 operated patients, 16 (4%) were excluded due to endoscopically, surgically, or radiologically proven anastomotic leakage, leaving 397 patients for final analysis. Fifty-four patients (14%) developed SSI. Of these, 21 (39%) presented with incisional SSI, while 33 (61%) were diagnosed with organ space infection.

Baseline characteristics and diagnoses are displayed in Table 1. Both groups (SSI versus no SSI) were comparable except for smoking status (22 versus 18%, *p* = 0.051) and previous abdominal surgery (46 versus 30%, *p* = 0.005).

Surgical details are illustrated in Table 2. Univariate risk factors for SSI were emergency setting (50 versus 27%, *p* < 0.001) and wound class as assessed by the independent surveillance committee (III-IV: 65 versus 51%, *p* = 0.015), while minimally invasive surgery consisted a protective factor (41 versus 72%, *p* < 0.001).

### 3.2. ERAS Compliance and Modifiable Pre- and Perioperative ERAS Items

**Figure 1:**
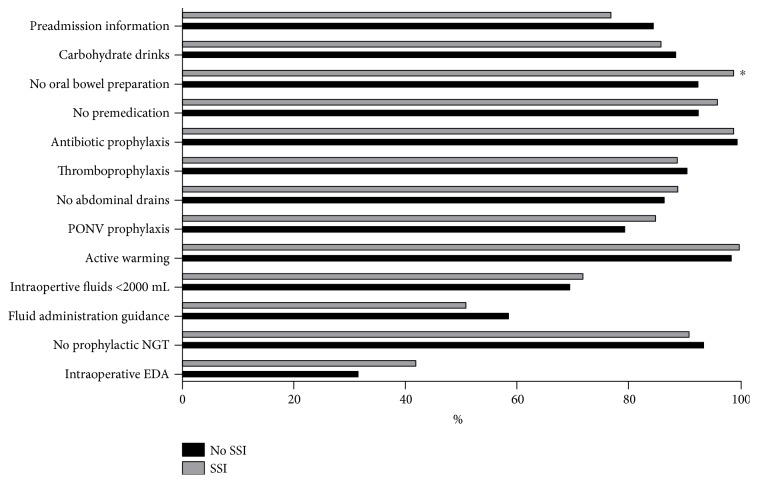
Pre- and intraoperative modifiable ERAS items. Comparison of compliance to modifiable pre- and intraoperative ERAS-related items among patients with SSI (black bars) and patients without SSI (grey bars). Premedication = administration of long-acting sedative medication. SSI: surgical site infection; PONV: postoperative nausea and vomiting; EDA: epidural analgesia; NGT: nasogastric tube. ∗ indicates statistical significance (*p* < 0.05).

Two hundred and eight patients (52%) presented an overall compliance of at least 70% to ERAS items. Compliance of >70% was linked to an apparent decreased SSI rate, which did not reach the limit of significance after univariate analysis (41 versus 54%, *p* = 0.065). Modifiable pre- and perioperative ERAS items are illustrated in Figure 1. All items were comparable between the two groups except for oral bowel preparation (7 versus 1%, *p* = 0.014).

Cefuroxim/metronidazole administration within one hour of incision was comparable between the two groups (63 versus 72%, *p* = 0.174).

### 3.3. Independent Risk Factors for SSI

**Figure 2:**
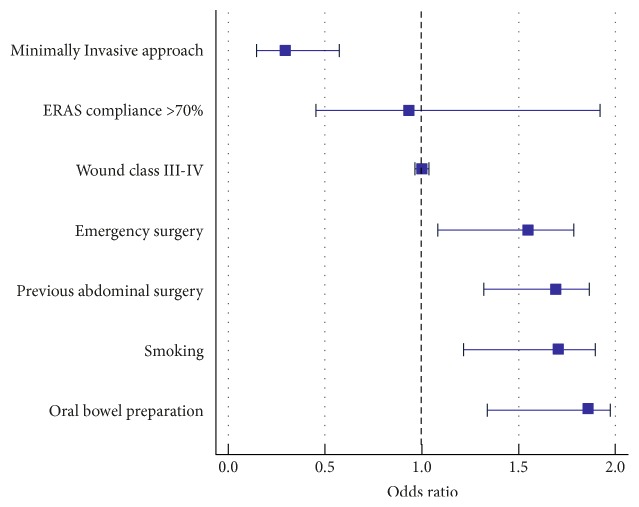
Independent risk factors for SSI. Odds ratio for outcome SSI, 95% confidence IntervaI.

Independent risk factors for SSI were emergency surgery (OR 1.56; 95% CI 1.09–1.78, *p* = 0.026), previous abdominal surgery (OR 1.7; 95% CI 1.32–1.87, *p* = 0.004), smoking (OR 1.71; 95% CI 1.22–1.89, *p* = 0.014), and oral bowel preparation (OR 1.86; 95% CI 1.34–1.97, *p* = 0.013), while minimally invasive surgery (OR 0.3; 95% CI 0.16–0.56, *p* < 0.001) protected against SSI. Compliance to ERAS items of >70% was not a protective factor for SSI after multivariate analysis (OR 0.94; 95% CI 0.46–1.92, *p* = 0.86).  Figure 2 gives an overview of independent factors associated with SSI.

### 3.4. Outcome

SSI were associated with longer mean length of hospital stay (20 ± 17 versus 7 ± 6 days, *p* < 0.001) and higher readmission rate (24 versus 2%, *p* < 0.001).

## 4. Discussion

In the present study of 397 colorectal patients, it was not possible to demonstrate a benefit of ERAS compliance on SSI incidence, while minimally invasive surgery was clearly protective.

Fourteen percent of patients after colectomy presented with SSI within a 30-day postoperative observation period by an independent National surveillance committee. Several risk factors including smoking, emergency surgery, open surgery, and previous abdominal surgery were independently associated with SSI. According to ERAS guidelines, oral bowel preparation was not recommended for colonic surgery and was carried out in only 2% of the present cohort almost exclusively in the early study period. Interestingly, this was the only modifiable ERAS item independently associated with an increased SSI rate. Overall compliance to ERAS pathway of >70% was not linked to a significant decrease in SSI neither in uni- nor in multivariate analysis.

A recent meta-analysis assessed the impact of enhanced recovery protocols on healthcare-related infections in patients undergoing abdominal and pelvic surgeries [18]. Interestingly, out of 36 included randomized controlled trials, only one single study observed a beneficial effect of ERAS. However, meta-analysis of pooled data led to a risk reduction of 25% comparing ERAS to traditional care. This finding joins formerly described beneficial effects of ERAS compared to traditional care. Reduced overall morbidity, length of stay, and costs have been repeatedly proven as a result of decreased surgical stress response [2, 14, 19]. In the present study, all patients without exception were treated within a standardized ERAS care pathway, including emergency operations. The aim was thus not to compare SSI rate to traditional care but to assess SSI rate among different compliance groups. Gustafsson has shown that best results were achieved with the highest overall compliance to ERAS [15]. These results have been confirmed in a recent study suggesting a minimal overall compliance of 70% to achieve improved postsurgical results [16]. As a consequence, the present study aimed to compare compliant (>70%) to less compliant (<70%) patients and to analyze individual compliance to modifiable ERAS items. Two reasons might account for similar SSI rates among compliant and less compliant patients. First, the modest sample size might be prone to type II error. Second, ERAS has been shown to decrease above all nonsurgical, especially cardiopulmonary complications, rather than surgical complications [2].

In the present cohort, 14% of patients were diagnosed with either superficial or organ space SSI as defined. This rate is comparable to former large-scale studies [20–22]. It has to be emphasized that all patients were systematically followed by dedicated and independent abstractors limiting artificially low infection rates due to detection bias.

Surgical complications and anastomotic leaks are mainly linked to patient-related factors or caused by technical shortcomings. Therefore, surgical complications were shown not to be modified by ERAS pathways [2]. Inevitably, anastomotic leaks entail intra-abdominal abscess and frequently superficial side infection as a consequence. Hence, patients with a proven anastomotic leak were excluded as an overwhelming confounder for the purpose of the present study. However, contamination class assessed by the treating surgeon and independent assessment of perioperative contamination by the surveillance committee (NNIS score, wound class) were analyzed as potential risk factors. Despite significance after univariate analysis, wound class did not correlate with SSI after multivariate analysis. An explanation might be that both measures rely on subjective assessment, and hence observer-dependent differences in individual grading of contamination might be a consequence. Interestingly, surgeons classified most procedures (88%) as contamination class II, while half of the procedures were classified as grades III-IV by the independent committee. Another parameter that deserves in-depth discussion is antimicrobial prophylaxis [23]. In the present cohort, antibiotic prophylaxis was delivered in all patients without exception, however with appropriate timing of administration between 30–60 min before incision in 71% of patients only. Despite a difference of almost 10% among the two groups (63 versus 72%), this result was not significant, possibly due to type II error. While timing of antibiotic administration is crucial, mode of administration is debated [24]. While a combined oral and IV administration has been most beneficial in a former study [25], no superiority of this combination was observed in a more recent randomized trial [26]. Due to an inherent risk of dehydration, distress and postoperative ileus, mechanical bowel preparation is not recommended by ERAS guidelines [3], in line with a comprehensive Cochrane review of almost 6000 patients [27], and should remain an exception, that is, when intraoperative colonoscopy is needed. Whether oral antibiotics need to be combined with mechanical bowel preparation is a matter of debate [24]. Recent evidence suggested this combination [28–30] as a way to reduce SSI, in contrast to the results of former randomized studies [31]. To draw final conclusions, results of ongoing randomized trials (i.e., MOBILE trial, NCT02652637) are eagerly awaited before the next revision of enhanced recovery guidelines for colorectal surgery.

In a large quality improvement project including more than 27000 patients who underwent colonic surgery, open approach and active smoking were independent risk factors for both superficial and deep/organ space infection [32], similar to the results of the present study. It is therefore important to insist during the outpatient visit on smoking cessation prior to surgery. Minimally invasive colonic surgery was associated with a lower rate of SSI in several studies [33, 34]. Moreover, the combination of laparoscopy and ERAS might be even more beneficial [35–37]. Several known risk factors for SSI, including diabetes and hypothermia, were not retained in the present study [38, 39], probably reflecting benefits of the ERAS protocol including stringent carbohydrate homeostasis and high compliance to hypothermia prevention.

Some limitations of the present study beyond its retrospective nature need to be discussed. Due to modest sample size, the study is prone to type II error. Some of the analyzed items (wound/contamination class and NNIS score) rely on subjective assessment. Moreover, it is delicate to compare SSI rate to previous reports since definition of SSI was not standardized among studies. However, the strength of the present study was independent and prospective assessment of SSI in all patients by the official National surveillance committee. Thus, the results are likely to be representative for everyday practice for physicians performing colonic surgery within an ERAS program.

## 5. Conclusions

In conclusion, SSI remain a frequent problem after colonic surgery. While ERAS compliance had no independent impact, open approach, emergency surgery, smoking, and bowel preparation were identified as independent risk factors for SSI. Preoperative smoking cessation and favoring of minimally invasive surgery might contribute to better outcomes.

## Figures and Tables

**Table 1:** Baseline characteristics.

	All patients (*n* = 397)	SSI (*n* = 54)	No SSI (*n* = 343)	*p*
Age (years) (mean ± SD)	64 ± 16	66 ± 15	64 ± 16	0.395
*Gender*				
Male (%)	186 (47)	24 (44)	162 (47)	0.703
*BMI (kg/m2)*				
>25 (%)	212 (53)	31 (57)	181 (53)	0.525
*ASA group*				
I-II (%)	258 (65)	35 (65)	223 (65)	0.977
III-IV (%)	139 (35)	19 (35)	120 (33)	
Smoking (%)	75 (19)	12 (22)	63 (18)	0.051
Diabetes (%)	48 (13)	6 (11)	42 (12)	0.765
Immunosuppression (%)	47 (12)	5 (9)	42 (12)	0.528
Neoadjuvant chemotherapy (%)	26 (7)	3 (6)	24 (7)	0.696
Previous abdominal surgery (%)	129 (33)	25 (46)	104 (30)	**0.005**
Malignancy (%)	210 (53)	35 (65)	175 (51)	0.215
*Diagnosis (%)*				
Primary adenocarcinoma	179 (45)	18 (33)	161 (47)	0.491
Other primary malignancy	7 (2)	1 (2)	6 (2)	
Metastatic disease	13 (3)	4 (7)	9 (2)	
Benign tumor/polyp	22 (6)	2 (4)	20 (6)	
Inflammatory bowel disease	26 (6)	5 (9)	21 (6)	
Diverticular disease	90 (23)	15 (28)	75 (22)	
Functional disorder	28 (7)	4 (7)	24 (7)	
Other benign disorder	32 (8)	5 (10)	27 (8)	

SSI: surgical site infection; BMI: body mass index; ASA: American Society of Anaesthesiologists. Significant values are indicated in bold characters.

**Table 2:** Surgical details.

	All patients (*n* = 397) (%)	SSI (*n* = 54) (%)	No SSI (*n* = 343) (%)	*p*
*Procedure*				
Left colectomy	46 (12)	7 (13)	39 (11)	0.519
Sigmoid resection	140 (35)	17 (32)	123 (36)	
Right colectomy	135 (34)	21 (39)	114 (33)	
Total colectomy	28 (7)	5 (9)	23 (7)	
Hartmann reversal	39 (10)	4 (7)	35 (10)	
Other	9 (2)	0	9 (3)	
Minimally invasive surgery	270 (68)	22 (41)	248 (72)	**<0.001**
Emergency	118 (30)	27 (50)	91 (27)	**<0.001**
Operating time > 3 hours	186 (47)	28 (52)	158 (46)	0.465
*New stoma*				
No	340 (86)	50 (92)	290 (85)	0.293
Ileostomy	28 (7)	2 (4)	26 (7)	
Colostomy	29 (7)	2 (4)	27 (8)	
*Anastomotic technique*				
Hand-sewn	74 (18)	10 (19)	64 (19)	0.73
Circular staplers	177 (45)	21 (39)	156 (46)	
Linear staplers	103 (26)	18 (33)	85 (25)	
Other/no anastomosis	43 (11)	5 (9)	38 (8)	
*Contamination class (assessed by surgeon)*				
II	351 (88)	50 (92)	301 (88)	0.312
III-IV	46 (12)	4 (8)	42 (12)	
*Wound class (assessed by surveillance committee)*				
II	187 (47)	19 (35)	168 (49)	**0.015**
III-IV	210 (53)	35 (65)	175 (51)	
*Antibiotic administration*				
<1 hour before incision	281 (71)	34 (63)	247 (72)	0.174
*NNIS score*				
0	74 (19)	7 (13)	67 (20)	0.059
1	145 (37)	15 (28)	130 (38)	
2	138 (35)	22 (41)	116 (34)	
3	40 (10)	10 (19)	30 (9)	

SSI: surgical site infection; Contamination/wound class: II—clean contaminated, III—contaminated, IV—infectious; NNIS: National Nosocomial Infection Surveillance. Significant values are indicated in bold characters.
